# Quartic Regularity

**DOI:** 10.1007/s10013-024-00720-z

**Published:** 2025-03-12

**Authors:** Yurii Nesterov

**Affiliations:** 1 Corvinus Centre for Operations Research (CCOR) at Corvinus University of Budapest, Budapest, Hungary; 2 Emeritus professor at Catholic University of Louvain (UCLouvain), 1348 Louvain-la-Neuve, Belgium

**Keywords:** Composite convex minimization, Second-order methods, Global complexity bounds, High-order proximal-point methods, 65K15, 90C33

## Abstract

In this paper, we propose new linearly convergent second-order methods for minimizing convex quartic polynomials. This framework is applied for designing optimization schemes, which can solve general convex problems satisfying a new condition of quartic regularity. It assumes positive definiteness and boundedness of the fourth derivative of the objective function. For such problems, an appropriate quartic regularization of Damped Newton Method has global linear rate of convergence. We discuss several important consequences of this result. In particular, it can be used for constructing new second-order methods in the framework of high-order proximal-point schemes (Nesterov, Math. Program. **197**, 1–26, 2023 and Nesterov, SIAM J. Optim. **31**, 2807–2828, 2021). These methods have convergence rate $\tilde{O}(k^{-p})$, where *k* is the iteration counter, *p* is equal to 3, 4, or 5, and tilde indicates the presence of logarithmic factors in the complexity bounds for the auxiliary problems, which are solved at each iteration of the schemes.

## Introduction

### Motivation

After appearance in 2006 of the first second-order method with global efficiency bounds [22], the question of finding the right framework for complexity analysis of the higher-order schemes became one of the most interesting research directions in Convex Optimization. Indeed, the performance estimates of Cubic Newton Method [22] and its accelerated version [16] clearly demonstrated that the existing classification of complexity of optimization problems in accordance to the *condition number* of the objective^1^ fits well only the first-order schemes. The higher-order methods are insensitive to this characteristics since, in some sense, the influence of the quadratic part of the objective is eliminated by a single iteration of the simplest second-order scheme. Thus, it became necessary to find new characteristics helping in ranking the performance of the new methods.

The first attempt of introducing a notion of the second-order non-degeneracy was done already in [16]. In the complexity analysis of [22], the assumption for bounding the growth of the objective was taken from the classical result of Kantorovich [14] on local quadratic convergence of Newton Method. This was the Lipschitz continuity of the Hessian of the objective function. However, in [16], the lower bound for the growth of the objective was ensured by uniform convexity of *degree three*. The justification of this combination was purely algebraic: under these assumptions it was possible to prove the global *linear* rate of converge of the second-order methods. As compared to the theory of first-order methods, this choice looks much less adequate since it is not supported by any regular source of such problems, related to the real-life applications.

Still, during the next decade, the development of the second-order methods was accomplished within this framework. For completeness of the picture, we mention interesting results on different variants of Cubic Regularization [7, 8], universal methods [13], accelerated second-order methods based on contractions [9, 11], and many others.

However, very soon it was shown that for convex functions we can efficiently implement also the higher-order optimization methods [17]. This result justifies an extensive study of these schemes [6, 10, 12, 19], including the lower complexity bounds [1, 4]. In particular, it was shown that the methods of the order three and higher have much faster rate of convergence than the best possible rate of the second-order schemes (on their “natural” problem classes).

The latter fact explains the level of the interest caused by an observation that practically all third-order methods can be implemented by a proper use of the second-order oracle, preserving at the same time their higher rate of convergence [18, 20, 21]. The first results of this type were based on the difference approximation of the product of the third-order derivative by two vectors [18], while the other papers employ the framework of the high-order proximal-point operators.

These results reveal the question on a proper attribution of optimization methods to particular problem classes. This link can be justified only by comparing the rates of convergence of the optimization schemes with the lower complexity bounds. Today it is clear that the initial naive hope that every problem class (formed by functions with uniformly bounded derivative of certain degree) can be unanimously related to the methods of certain order is too optimistic. This is may be true for the first-order methods, traditionally assigned to functions with bounded second derivatives. However, the next step in this classification is already much more involved.

Indeed, if we agree that the natural problem class for the second-order methods is formed by functions with bounded third derivative, then we must accept the corresponding bound on the maximal possible global rate of convergence in functional residual, which is of the order $O(k^{-3.5})$, with *k* being the number of calls of oracle [4]. On the other hand, as it was shown in [17], the third derivative of convex functions is *not* an independent characteristic. It can be estimated from above by a combination of the second and the fourth derivatives. And if we assume instead the boundedness of these two derivatives, then the limits in the rate of convergence are relaxed up to $O(k^{-5})$.

From the view point of global efficiency bounds, this perspective is very interesting. One possible way of implementing this idea was already presented in the papers [20, 21]. The corresponding approach is based on the notion of the high-order proximal operators, approximated by auxiliary minimization schemes guided by the relative smoothness condition [5, 15]. In this paper, we develop a more direct approach based on the bounds for the *fourth derivative*. Our main observation is that the fourth derivative (contrary to the third one) can be *positive semidefinite*, and this property helps in further acceleration of optimization schemes.

### Contents

In Section 2, we consider the problem of minimizing convex quartic polynomial in composite setting. We show that the corresponding quartic form is always convex and study its properties. In the next Section 3, we present the Damped Quartic Newton Method (DQNM), which regularizes the quadratic approximation of the objective by the fourth power of the norm, defined by the forth derivative. We prove that the corresponding global rate of convergence is linear with the absolute reduction factor. In Section 4, we show that the regularization norm in DQNM can be replaced by a simpler one. The rate of convergence of the corresponding scheme is still linear, but it depends on the condition number of the new norm with respect to the optimal one. Note that the corresponding variant of DQNM is essentially a second-order method.

In Section 5, we present a method for solving a composite optimization problem, where the smooth part of the objective is *quartic regular*. This means that its fourth-order derivative is uniformly positive definite and bounded on the domain of the composite term. We show that such a function admits global upper and lower bounds for their growth with respect to local approximations. Using the corresponding inequalities, we justify a linear rate of convergence of some variant of DQNM.

In Section 6, we present some applications of our results. First of all, we show that DQNM can be applied to functions with positive-semidefinite fourth derivative. In terms of the number of calls of oracle, it has the rate of convergence $\tilde{O}(k^{-3})$. In the remaining part of the section we discuss applications of DQNM to the framework of proximal point methods. We show that it can be used for designing optimization methods with the rate of convergence $\tilde{O}(k^{-p})$ with $p \in \{3,4,5\}$. All these methods are of the order two.

### Notation

Let us fix a finite-dimensional real vector space $\mathbb {E}$. We measure distances in $\mathbb {E}$ by a general norm $\Vert \cdot \Vert$. Its dual space is denoted by $\mathbb {E}^*$. It is the space of all linear functions on $\mathbb {E}$, for which we define the norm in the standard way:

$$ \Vert g\Vert ^*= \max _{x \in \mathbb {E}} \{\langle g, x \rangle : \; \Vert x \Vert \le 1\}, \qquad g \in \mathbb {E}^*. $$

Using this norm, we can define an induced norm for a self-adjoint linear operator $A: \mathbb {E} \rightarrow \mathbb {E}^*$ as follows:

$$ \Vert A\Vert = \max _{x \in \mathbb {E}} \{|\langle A x, x \rangle | : \; \Vert x \Vert \le 1 \}. $$

For defining Euclidean norm, we introduce a positive-definite self-adjoint linear operator $B: \mathbb {E} \rightarrow \mathbb {E}^*$ (notation $B \succ 0$). Then for $x \in \mathbb {E}$ and $g \in \mathbb {E}^*$ we have

$$ \Vert x\Vert _B = \langle Bx, x \rangle ^{1/2}, \qquad \Vert g\Vert _{B}^*= \langle g, B^{-1} g \rangle ^{1/2}. $$

If no ambiguity arise, we drop index *B* in this notation. Note that for any self-adjoint linear operator $A: \mathbb {E} \rightarrow \mathbb {E}^*$ we have

$$ \Vert A \Vert = \min \{ \lambda : \; \lambda B \succeq \pm A \}. $$

If in addition, *A* is positive semidefinite and $L \ge \Vert A\Vert$, then

1.1 $$\begin{aligned} A B^{-1} A \preceq L A. \end{aligned}$$

Finally, for a linear operator $A: \mathbb {E} \rightarrow E_1$, we denote

$$ \sigma _{\min }(A) = \min _{\Vert x \Vert =1} \Vert A x \Vert , \qquad \sigma _{\max }(A) = \max _{\Vert x \Vert =1} \Vert A x \Vert . $$

For function $f(\cdot )$ with open domain $\textrm{dom}\,f \subseteq \mathbb {E}$, we denote by $\nabla f(x) \in \mathbb {E}^*$ its gradient and by $\nabla ^2 f(x): \mathbb {E} \rightarrow \mathbb {E}^*$ its Hessian at point $x \in \textrm{dom}\,f$. For the mixed directional derivative of order $p \ge 1$ along directions $h_1, \dots , h_p$ in $\mathbb {E}$, we use notation $D^pf(x)[h_1, \dots , h_p]$. Note that under the standard continuity assumptions this is a symmetric *p*-linear form. If all directions are the same, we use notation $D^pf(x)[h]^p$.

We use the same notation for symmetric 4-linear forms. Let $f[x_1,x_2,x_3,x_4]$ be such a form with $x_i \in \mathbb {E}$, $i=1,\dots ,4$. If all vectors are the same, we denote the value of this form by $f[x]^4$. This is a multivaried polynomial of degree four. Let us treat it as a function $\varphi (x) = f[x]^4$. Then the vector $f[x]^3 \in \mathbb {E}^*$ is defined as follows:

$$ f[x]^3 = \frac{1}{4} \nabla \varphi (x), \quad x \in \mathbb {E}. $$

Note that $\langle f[x]^3, x \rangle = f[x]^4$. Similarly,

$$ f[x]^2 = \frac{1}{12} \nabla ^2 \varphi (x), \quad x \in \mathbb {E}. $$

Then $f[x]^2[y]^2 = \frac{1}{12} \langle \nabla ^2\varphi (x) y, y \rangle$ and $f[x]^2[x]^2 = f[x]^4$. Finally,

$$ f[x] = \frac{1}{24} D^3\varphi (x), \quad x \in \mathbb {E}, $$

and $$f[x][y]^3 = \frac{1}{24} D^3\varphi (x) [y]^3$$, $$f[x][x]^3 = f[x]^4$$. Thus, $$f = \frac{1}{24} D^4\varphi $$.

Finally, we need one result, which is usually proved under more restrictive assumptions.

### Lemma 1

Let function $f(\cdot )$ be convex and twice continuously differentiable at its open domain. For two points $x, y \in \textrm{dom}\,f$, denote $M_{x,y} = \max _{0\le \alpha \le 1} \Vert \nabla ^2 f(\alpha x + (1-\alpha )y) \Vert$, where the norm is Euclidean. Then

1.2 $$\begin{aligned} \Vert \nabla f(x) - \nabla f(y)\Vert _B^*\le M_{x,y} \langle \nabla f(x) - \nabla f(y), x - y \rangle . \end{aligned}$$

### Proof

Denote $G = \int _0^1 \nabla ^2 f(x + \tau (y - x)) d \tau$. Then $G(y-x) = \nabla f(y) - \nabla f(x)$ and $\Vert G \Vert \le M_{x,y}$. Therefore, inequality (1.2) follows from (1.1).$\square$

## Positive Semidefinite Quartic Forms

In the first part of this paper, we are interested in numerical methods for finding an approximate solution to the following minimization problem:

2.1 $$\begin{aligned} F^*= \min _{x \in \textrm{dom}\,\psi } \left[ F(x) = f(x) + \psi (x)\right] , \end{aligned}$$

where $\psi (\cdot )$ is a *simple* closed convex function and $f(\cdot )$ is a convex *quartic polynomial*. Since, $f(\cdot )$ is a polynomial of degree four, its Taylor expansion with respect to any point in $\mathbb {E}$ is exact. Namely, for any $x, y \in \mathbb {E}$ we have

2.2 $$\begin{aligned} f(y)= &   f(x) + \langle \nabla f(x), y-x] \rangle + \frac{1}{2} \langle \nabla ^2 f(x)(y-x), y-x \rangle \nonumber \\  &   + \frac{1}{6} D^3f(x)[y-x]^3 + f_4[y-x]^4, \end{aligned}$$

where $f_4[\cdot ,\cdot ,\cdot , \cdot ]$ is a symmetric four-linear form in $\mathbb {E}$. Thus, $f_4 = \frac{1}{24} D^4f$.

Convexity of function $f(\cdot )$ has several important consequences.

### Theorem 1

For any *x* and *y* in $\mathbb {E}$ we have

2.3 $$\begin{aligned} 0 \le f_4[x]^2[y]^2. \end{aligned}$$

This property implies the following relations:

2.4 $$\begin{aligned} 2 f_4[x,y]\preceq &   f_4[x]^2 + f_4[y]^2,\end{aligned}$$

2.5 $$\begin{aligned} (f_4[x]^2[y]^2)^2\le &   f_4[x]^4 \cdot f_4[y]^4,\end{aligned}$$

2.6 $$\begin{aligned} (f_4[x]^3[y])^2\le &   f_4[x]^4 \cdot f_4[x]^2[y]^2. \end{aligned}$$

### Proof

Indeed, taking in (2.2) $x = 0$, we have $\nabla ^2 f(y) = \nabla ^2 f(0) + D^3 f(0)[y] + \frac{1}{2} D^4 f(0)[y]^2$. Therefore,

$$ 0 \le \langle \nabla ^2 f(y) h, h \rangle = \langle \nabla ^2 f(0) h, h \rangle + D^3 f(0)[y][h]^2 + 12 f_4[y]^2[h]^2. $$

Replacing now *y* by $\tau y$ and taking the limit as $\tau \rightarrow \infty$, we get inequality (2.3). It implies that $f_4[x-y]^2 \succeq 0$. And this is exactly the relation (2.4).

Furthermore, in view of (2.3), for any $\tau \in \mathbb {R}$ we have $f_4[x-\tau y]^2[x+\tau y]^2 \ge 0$. If we open the brackets, then we get

$$ 0 \le f_4[x]^4 + \tau ^4 f_4[y]^4 - 2 \tau ^2 f_4[x]^2[y]^2. $$

If $f_4[y]^4 = 0$, then $f_4[x]^2[y]^2 = 0$ and (2.5) holds. If not, then by minimizing the right-hand side of the latter inequality in $\tau$, we get the relation (2.5). Finally, minimizing the right-hand side of inequality

$$ 0 \le f_4[x]^2[x - \tau y ]^2 $$

in $\tau$, we get inequality (2.6). $\square$

Define $d(x) = f_4[x]^4$. Note that

$$\begin{aligned} d(tx) = |t|^4 d(x), \quad x \in \mathbb {E},~ t \in \mathbb {R}. \end{aligned}$$

Let us look at some properties of this function. First of all, note that all derivatives of this function can be written in terms of the form $f_4$ as follows:

2.7 $$\begin{aligned} \begin{array}{rclrcl} \langle \nabla d(x), h \rangle &  = &  4 f_4[x]^3[h], \quad \langle \nabla ^2 d(x) h, h \rangle &  = &  12 f_4[x]^2[h]^2,\\ D^3d(x)[h]^3 &  = &  24 f_4[x][h]^3, \quad D^4 d(x)[h]^4 &  = &  24 f_4[h]^4, \end{array} \end{aligned}$$

where *x* and *h* are arbitrary vectors in $\mathbb {E}$. Thus, by inequality (2.3), we can see that function $d(\cdot )$ is convex.

Let us assume now that the form $f_4$ is positive definite:

2.8 $$\begin{aligned} f_4[x]^4 > 0 \quad \forall x \in \mathbb {E} \setminus \{0\}. \end{aligned}$$

Then, we can define in $\mathbb {E}$ the following norm:

$$\begin{aligned} \Vert x\Vert _f = [d(x)]^{1/4}, \quad x \in \mathbb {E}. \end{aligned}$$

This norm has several interesting properties. First of all, it justifies the uniform convexity of function $d(\cdot )$ itself.

### Lemma 2

Function $d(\cdot )$ is uniformly convex of degree four with constant $\sigma _d = \frac{4}{3}$:

$$\begin{aligned} d(y) \ge d(x) + \langle d(x), y - x \rangle + \frac{1}{4} \sigma _d \Vert y - x \Vert _f^4, \quad x, y \in \mathbb {E}. \end{aligned}$$

Moreover, we have the following lower quadratic bound for the growth of function $d(\cdot )$:

2.9 $$\begin{aligned} d(y) \ge d(x) + \langle d(x), y - x \rangle + \frac{1}{6} \langle \nabla ^2d(x)(y-x), y - x \rangle , \quad x, y \in \mathbb {E}. \end{aligned}$$

### Proof

Indeed, for any *x* and *y* from $\mathbb {E}$, we have

$$\begin{aligned} d(y) - d(x) - \langle d(x), y - x \rangle= &   \frac{1}{2} \langle \nabla ^2 d(x) (y-x), y- x \rangle \\  &   + \frac{1}{6} D^3d(x)[y-x]^3 + \frac{1}{24} D^4d(x)[y-x]^4\\\overset{(2.7)}{=} &   6 f_4[x]^2[y-x]^2 + 4 f_4[x][y-x]^3 + f_4[y-x]^4\\\overset{(2.6)}{\ge } &   6 f_4[x]^2[y-x]^2 \!-\! 4 \left( f_4[y\!-\!x]^4 \cdot f_4[x]^2[y\!-\!x]^2 \right) ^{1/2}\!\! +\! f_4[y\!-\!x]^4. \end{aligned}$$

Minimizing the right-hand side of this inequality in $f_4[x]^2[y-x]^2$, we get

$$ d(y)- d(x) - \langle d(x), y - x \rangle \ge \left( 1 - \frac{2}{3}\right) f_4[y-x]^4 = \frac{1}{3} \Vert y - x \Vert ^4_f. $$

Finally, minimizing the same inequality in $f_4[y-x]^4$, we get

$$ d(y)- d(x) - \langle d(x), y - x \rangle \ge (6 - 4) f_4[x]^2[y-x]^2, $$

and this is (2.9). $\square$

Consider now the function $Q_f(x) = \frac{1}{2} \Vert x \Vert ^2_f$.

### Lemma 3

Function $Q_f(\cdot )$ is strongly convex and has Lipschitz continuous gradients with respect to the norm $\Vert \cdot \Vert _f$:

$$\begin{aligned} \frac{1}{12 \Vert x\Vert _f^2} \langle \nabla ^2 d(x)h, h \rangle \le \langle \nabla ^2 Q_f(x)h,h\rangle \le 3 \Vert h\Vert ^2_f, \quad x, h \in \mathbb {E}. \end{aligned}$$

### Proof

Since $Q_f(x) = \frac{1}{2} d^{1/2}(x)$, for any *x* and *h* from $\mathbb {E}$, we have

$$\begin{aligned} \langle \nabla Q_f(x), h \rangle= &   d^{-1/2}(x) f_4(x)[x]^3[h],\\ \langle \nabla ^2 Q_f(x)h, h \rangle= &   3 d^{-1/2}(x) f_4(x)[x]^2[h]^2 - 2 d^{-3/2}(x) (f_4[x]^3[h])^2\\\overset{(2.6)}{\ge } &   d^{-1/2}(x) f_4(x)[x]^2[h]^2. \end{aligned}$$

On the other hand,

$$ \langle \nabla ^2 Q_f(x)h, h \rangle \le 3 d^{-1/2}(x) f_4(x)[x]^2[h]^2 \overset{(2.5)}{\le } 3 \Vert h\Vert _f^2. $$

$$\square $$

## Minimizing Convex Polynomials of Degree Four

In this section, we are going to check which properties of function $d(\cdot )$ are inherited by function $f(\cdot )$ and how we can use them for constructing minimization schemes. The next statement is a variant of Theorem 1 in [17].

### Lemma 4

For any point *x*, direction $h \in \mathbb {E}$, and $\tau > 0$, we have

3.1 $$\begin{aligned} D^3f(x)[h] \preceq \frac{1}{\tau } \nabla ^2 f(x) + 12 \tau f_4[h]^2. \end{aligned}$$

### Proof

Since $f(\cdot )$ is a convex quartic polynomial, for any $\tau > 0$ we have

$$ 0 \preceq \nabla ^2f(x - \tau h) = \nabla ^2f(x) - \tau D^3f(x)[h] + \frac{\tau ^2}{2} D^4f(x)[h]^2. $$

Dividing this inequality by $\tau$, we get

$$ D^3f(x)[h] \preceq \frac{1}{\tau } \nabla ^2f(x) + \frac{\tau }{2} D^4 f(x)[h]^2, $$

and this is the relation (3.1). $\square$

### Corollary 1

For all $x, y \in \mathbb {E}$ and $\tau > 0$, we have

3.2 $$\begin{aligned}  &   f(y) - f(x) - \langle \nabla f(x), y - x \rangle - \frac{1}{2} \langle \nabla ^2 f(x) (y-x), y - x \rangle \nonumber \\  &   \qquad \le \frac{1}{6 \tau } \langle \nabla ^2 f(x)(y-x), y - x \rangle + (1+2\tau ) \Vert y - x \Vert ^4_f,\end{aligned}$$

3.3 $$\begin{aligned}  &   f(y) - f(x) - \langle \nabla f(x), y - x \rangle - \frac{1}{2} \langle \nabla ^2 f(x) (y-x), y - x \rangle \nonumber \\  &   \qquad \ge - \frac{1}{6 \tau } \langle \nabla ^2 f(x)(y-x), y - x \rangle + (1-2\tau ) \Vert y - x \Vert ^4_f. \end{aligned}$$

### Proof

In view of relation (3.1), we have $|D^3f(x)[h]^3 |\le \frac{1}{\tau } \langle \nabla ^2f(x)h,h \rangle + 12 \tau \Vert h \Vert ^4_f$. Therefore,

$$\begin{aligned}  &   f(y) - f(x) - \langle \nabla f(x), y - x \rangle - \frac{1}{2} \langle \nabla ^2 f(x) (y-x), y - x \rangle \\  &   \quad \overset{(2.2)}{=} \frac{1}{6} D^3f(x)[y-x]^3 + \Vert y - x\Vert ^4_f \le \frac{1}{6 \tau } \langle \nabla ^2f(x)h,h \rangle + (1 + 2\tau ) \Vert h \Vert ^4_f. \end{aligned}$$

The second inequality can be justified in the same way.$\square$

Using inequalities (3.2) and (3.3), we can write down the lower and upper approximations for the smooth part of the objective function:

3.4a $$\begin{aligned} \bar{f}_{x,\tau }(y)= &   f(x) + \langle \nabla f(x), y - x \rangle + \frac{3\tau - 1}{6 \tau } \langle \nabla ^2 f(x) (y-x), y - x \rangle \nonumber \\  &   + (1- 2\tau ) \Vert y - x \Vert ^4_f \le f(y),\end{aligned}$$

3.4b $$\begin{aligned} \hat{f}_{x,\tau }(y)= &   f(x) + \langle \nabla f(x), y - x \rangle + \frac{1 + 3\tau }{6 \tau } \langle \nabla ^2 f(x) (y-x), y - x \rangle \nonumber \\  &   + (1+2\tau ) \Vert y - x \Vert ^4_f \ge f(y), \quad x, y \in \mathbb {E}. \end{aligned}$$

Note that we are interested in $\tau \in \left[ \frac{1}{3}, \frac{1}{2} \right]$.

Let us assume that the norm $\Vert \cdot \Vert _f$ is simple enough for minimizing the following auxiliary function,

$$ \hat{f}_{x,\tau }(y) + \psi (y), \quad y \in \textrm{dom}\,\psi , $$

in a closed form. Thus, we can use it for justifying methods for solving the problem (2.1).

Let us define the following parametric function:

3.5 $$\begin{aligned} \bar{f}_{x,\tau }(\alpha ,y)= &   f(x) + \langle \nabla f(x), y - x \rangle + \frac{3\tau - 1}{6 \tau \alpha } \langle \nabla ^2 f(x) (y-x), y - x \rangle \nonumber \\  &   + \frac{1- 2\tau }{\alpha ^3} \Vert y - x \Vert ^4_f, \quad y \in \mathbb {E}, \; \alpha > 0. \end{aligned}$$

Note that $\bar{f}_{x,\tau }(1,y) = \bar{f}_{x,\tau }(y) \le f(y)$ for all $y \in \mathbb {E}$. On the other hand, function $\bar{f}_{x,\tau }(\cdot ,\cdot )$ is jointly convex in its arguments. Therefore, the univariate function

$$ F^*_{x,\tau }(\alpha ) = \min _{y \in \textrm{dom}\,\psi } \left[ \bar{f}_{x,\tau }(\alpha ,y) + \psi (y)\right] , \quad \alpha > 0, $$

is convex. Let us prove the following result.

### Theorem 2

Let $\tau \in \left( \frac{1}{3}, \frac{1}{2}\right)$ and the parameter $\alpha > 0$ satisfies the following condition:

3.6 $$\begin{aligned} \alpha \le \min \left\{ \frac{3 \tau - 1}{3 \tau + 1}, \left[ \frac{1 - 2 \tau }{1 + 2 \tau } \right] ^{1/3} \right\} . \end{aligned}$$

Then

3.7 $$\begin{aligned} \min _{y \in \textrm{dom}\,\psi } \left[ \hat{f}_{x,\tau }(y) + \psi (y)\right] \le \alpha F^*+ (1-\alpha ) F(x). \end{aligned}$$

### Proof

Note that $F^*_{x,\tau }(1) \le F^*$. On the other hand, by continuity, we can define $F^*_{x,\tau }(0) = F(x)$. At the same time, condition (3.6) ensures the following relations:

$$ \frac{3 \tau - 1}{6 \tau \alpha } \ge \frac{3 \tau + 1}{6 \tau }, \quad \frac{1 - 2 \tau }{\alpha ^3} \ge 1 + 2 \tau . $$

This means that $\bar{f}_{x,\tau }(\alpha ,y) \ge \hat{f}_{x,\tau }(y)$ for all $y \in \mathbb {E}$. Hence,

$$\begin{aligned} \min _{y \in \textrm{dom}\,\psi } \left[ \hat{f}_{x,\tau }(y) + \psi (y)\right]\le &   F^*_{x,\tau }(\alpha ) \le \alpha F^*_{x,\tau }(1) + (1 - \alpha ) F^*_{x,\tau }(0) \\\le &   \alpha F^*+ (1-\alpha ) F(x). \end{aligned}$$

$$\square $$

Inequality (3.7) is important for justification of the following optimization scheme. Denote by $\tau _*$ the unique positive root of the following equation:

3.8 $$\begin{aligned} \frac{3 \tau - 1}{3 \tau + 1} = \left[ \frac{1 - 2 \tau }{1 + 2 \tau } \right] ^{1/3} . \end{aligned}$$

3.9 $$\begin{aligned} \begin{array}{c} {\textbf {Damped Quartic Newton Method}}\\ \\ \begin{array}{l} {{\textbf {Initialization.}}\, \textrm{Choose}\, x_0 \in \textrm{dom}\,\psi .}\\ \\ {{k {\textbf {th iteration}}\, (k \ge 0).}\, \textrm{Iterate}\, x_{k+1} = \arg \min \limits _{y \in \textrm{dom}\,\psi } \left[ \hat{f}_{x_k, \tau _*}(y) + \psi (y)\right] .}\\ \end{array} \end{array} \end{aligned}$$

### Theorem 3

For all $k \ge 0$, we have

$$\begin{aligned} F(x_k) - F^*\le (1 - \alpha _*)^k(F(x_0) - F^*), \end{aligned}$$

where $$\alpha ^*= \frac{3 \tau _*-1}{3 \tau _*+1} > 0.193$$ with $$\tau _*= \frac{1}{6}\sqrt{3 + \sqrt{33}}$$.

### Proof

Note that for any $x,y \in \mathbb {E}$ we have $\bar{f}_{x,\tau _*}(\alpha _*,y) = \hat{f}_{x,\tau _*}(y)$. Therefore,

$$ F(x_{k+1}) \overset{(3.4)}{\le } \hat{f}_{x_k,\tau _*}(x_{k+1}) + \psi (x_{k+1}) \overset{(3.7)}{\le } \alpha _*F^*+ (1- \alpha _*) F(x_k). $$

It remains to note that $\tau _*$ is the root of the equation

$$ (3 \tau -1)^3 (1+2\tau ) \overset{(3.8)}{=} (3 \tau +1)^3 (1-2\tau ). $$

After simplification, it is reduced to $54 \tau ^4 - 9 \tau ^2 = 1$, which gives us $\tau _*= \frac{1}{6}\sqrt{3 + \sqrt{33}}$. $\square$

Method (3.9) is implementable if the structure of the norm $\Vert \cdot \Vert _f$ is quite simple. Let us end this section with an important source of such problems.

### Example 1

For a convex function, consider its Taylor polynomials of degree three, augmented by a fourth power of Euclidean norm:

$$\begin{aligned} f_{\bar{x}, H}(y)= &   f(\bar{x}) + \langle \nabla f(\bar{x}), y - \bar{x} \rangle + \frac{1}{2} \langle \nabla ^2f(\bar{x})(y- \bar{x}), y - \bar{x}\rangle \\  &   + \frac{1}{6} D^3f(\bar{x})[y-\bar{x}]^3 + \frac{H}{24} \Vert y - \bar{x} \Vert ^4, \end{aligned}$$

where $\Vert \cdot \Vert$ is a Euclidean norm. The possibility to minimize this polynomial is essential for the third-order schemes. It can be proved [17] that for $H \ge 3 L_3$, where $L_3$ is the Lipschitz constant for third derivative of function $f(\cdot )$, the quartic polynomial $f_{\bar{x}, H}(\cdot )$ is convex. Note that it fits our framework since $D^4 f_{\bar{x}, H}(x)[h]^4 = H \Vert h \Vert ^4$. In this case, we can can replace the definition (3.5) by the following implicit form:

$$\begin{aligned} \bar{f}_{x,\tau }(\alpha ,y)= &   \max _{r\ge 0} \left\{ f(x) + \langle \nabla f(x), y - x \rangle + \frac{3\tau - 1}{6 \tau \alpha } \langle \nabla ^2 f(x) (y-x), y - x \rangle \right. \\  &   \left. + \frac{1- 2\tau }{\alpha ^3} \left[ 2r \Vert y - x \Vert ^2 - \frac{1}{2} r^2 \right] \right\} , \quad y \in \mathbb {E}, \; \alpha > 0. \end{aligned}$$

Then, in the important case $\psi (y) \equiv 0$, $y \in \mathbb {E}$, the auxiliary problem in method (3.9) can be solved by the standard tools of Linear Algebra. Indeed, we can exchange in this problem $\min _{ y \in \mathbb {E}}$ and $\max _{r > 0}$, minimize the internal quadratic function in $y \in \mathbb {E}$, and maximize the resulting univariate function in $r \ge 0$. $\square$

## Simplified Approximations

Let us introduce in the space $\mathbb {E}$ some norm $\Vert \cdot \Vert$, which is simpler than the norm $\Vert \cdot \Vert _f$. Since the space $\mathbb {E}$ is finite-dimensional, assumption (2.8) implies existence of two constants $0 < \mu \le L$ such that

4.1 $$\begin{aligned} \mu \Vert x \Vert ^4 \le f_4[x]^4 \le L \Vert x \Vert ^4, \quad x \in \mathbb {E}. \end{aligned}$$

In the particular case when $\Vert \cdot \Vert = \Vert \cdot \Vert _f$, we have $\mu = L = 1$. Denote by $q = \frac{\mu }{L}$ the condition number of the norm $\Vert \cdot \Vert$ with respect to the optimal norm $\Vert \cdot \Vert _f$.

Now, in view of (3.4), we can define the following simplified lower and upper bounds for the smooth part of the objective function:

4.2a $$\begin{aligned} \bar{\phi }_{x,\tau }(y)&\overset{\text {def}}{=}&f(x) + \langle \nabla f(x), y - x \rangle + \frac{3\tau - 1}{6 \tau } \langle \nabla ^2 f(x) (y-x), y - x \rangle \end{aligned}$$

4.2b $$\begin{aligned}  &   + (1- 2\tau ) \mu \Vert y - x \Vert ^4 \le f(y),\nonumber \\ \hat{\phi }_{x,\tau }(y)&\overset{\text {def}}{=}&f(x) + \langle \nabla f(x), y - x \rangle + \frac{1 + 3\tau }{6 \tau } \langle \nabla ^2 f(x) (y-x), y - x \rangle \\  &   + (1+2\tau ) L \Vert y - x \Vert ^4 \ge f(y), \quad x, y \in \mathbb {E}.\nonumber \end{aligned}$$

As in (3.5), define the following parametric functions

4.3a $$\begin{aligned} \bar{\phi }_{x,\tau }(\alpha , y)&\overset{\text {def}}{=}&f(x) + \langle \nabla f(x), y - x \rangle + \frac{3\tau - 1}{6 \tau \alpha } \cdot \langle \nabla ^2 f(x) (y-x), y - x \rangle \end{aligned}$$

4.3b $$\begin{aligned}  &   + (1- 2\tau ) \cdot \frac{\mu }{\alpha ^3} \Vert y - x \Vert ^4,\nonumber \\ \varPhi ^*_{x,\tau }(\alpha )= &   \min _{y \in \textrm{dom}\,\psi } \left[ \bar{\phi }_{x,\tau }(\alpha , y) + \psi (y) \right] , \quad \alpha > 0. \end{aligned}$$

Consider the following optimization method.

4.4 $$\begin{aligned} \begin{array}{c} {\textbf {Relaxed Quartic Newton Method}}\\ \\ \begin{array}{l} {{\textbf {Initialization.}}\, \textrm{Choose}\, x_0 \in \textrm{dom}\,\psi \, \textrm{and}\, \textrm{parameter}\, \tau \in \left( \frac{1}{3}, \frac{1}{2} \right) .}\\ \\ {{k {\textbf {th iteration}}\, (k \ge 0).}\, \textrm{Iterate}\, x_{k+1} = \arg \min \limits _{y \in \textrm{dom}\,\psi } \left[ \hat{\phi }_{x_k, \tau }(y) + \psi (y) \right] .}\\ \\ \end{array} \end{array} \end{aligned}$$

As compared with method (3.9), we use here a conservative approximation of the smooth part of the objective function: $\hat{\phi }_{x,\tau }(y) \overset{{(4.1)}}{\ge } \hat{f}_{x,\tau }(y)$ for all $x, y \in \mathbb {E}$.

### Theorem 4

Let the parameter $\tau$ of method (4.4) satisfy the following condition:

4.5 $$\begin{aligned} \alpha \le \min \left\{ \frac{3 \tau - 1}{3 \tau + 1} , \left[ q \cdot \frac{1 - 2 \tau }{1 + 2 \tau } \right] ^{1/3} \right\} \end{aligned}$$

for some $\alpha > 0$. Then, for all $k \ge 0$, we have

4.6 $$\begin{aligned} F(x_k) - F^*\le (1 - \alpha )^k(F(x_0) - F^*). \end{aligned}$$

### Proof

Indeed, in view of condition (4.5), we have

$$ \frac{3 \tau + 1}{6 \tau } \le \frac{3 \tau - 1}{6 \tau \alpha }, \quad (1 + 2 \tau ) L \le (1 - 2\tau ) \cdot \frac{ \mu }{\alpha ^3}. $$

Hence,

$$\begin{aligned} F(x_{k+1})\overset{(4.2)}{\le } &   \hat{\phi }_{x_k,\tau }(x_{k+1}) + \psi (x_{k+1}) \overset{(4.4)}{=} \min _{y \in \textrm{dom}\,\psi } \left[ \hat{\phi }_{x_k,\tau }(y) + \psi (y) \right] \\\overset{(4.5)}{\le } &   \min _{y \in \textrm{dom}\,\psi } \left[ \bar{\phi }_{x_k,\tau }(\alpha , y) + \psi (y) \right] \overset{(4.3)}{=} \varPhi ^*_{x_k,\tau }(\alpha ). \end{aligned}$$

On the other hand, since function $\varPhi ^*_{x_k,\tau }(\cdot )$ is convex, we have

$$ \varPhi ^*_{x_k,\tau }(\alpha ) \le \alpha \varPhi ^*_{x_k,\tau }(1) + (1 - \alpha ) \varPhi ^*_{x_k,\tau }(0) \overset{(4.2)}{\le } \alpha F^*+ (1 - \alpha ) F(x_k). $$

Thus, we obtain the bound (4.6). $\square$

Since we are interested in the maximal possible value of $\alpha$, let us try to understand which value of $\tau \in \left( \frac{1}{3}, \frac{1}{2} \right)$ makes the right-hand side of inequality (4.5) big. Note that

$$ \frac{3 \tau - 1}{3 \tau + 1} \ge \frac{2}{5} (3 \tau -1), \quad \tau \in \left( \frac{1}{3}, \frac{1}{2} \right) . $$

Let us look now at the function $\xi (\tau ) = \omega ^{1/3}(\tau )$ with $\omega (\tau ) = \frac{1 - 2 \tau }{1 + 2 \tau }$. Since

$$\begin{aligned} \xi '(\tau )= &   \frac{1}{3} \omega ^{-2/3}(\tau ) \omega '(\tau ), \qquad \xi ''(\tau ) = -\frac{2}{9} \omega ^{-5/3}(\tau ) (\omega '(\tau ))^2 + \frac{1}{3} \omega ^{-2/3} \omega ''(\tau ),\\ \omega '(\tau )= &   - \frac{4}{(1+2\tau )^2}, \quad \omega ''(\tau ) = \frac{16}{(1+2 \tau )^3}, \end{aligned}$$

we conclude that $\xi ''(\tau ) = \frac{16 \omega ^{-5/3}(\tau )}{9 (1+2 \tau )^4} (- 2 + 3(1-2 \tau ) ) \le 0$ for $\tau \in \left( \frac{1}{3}, \frac{1}{2} \right)$. Hence, for this interval, we have

$$ \xi (\tau ) \ge \xi \left( \frac{1}{3}\right) \cdot 3(1-2 \tau ) = 3(1-2 \tau ) \cdot 5^{-1/3}. $$

Thus, the condition (4.5) is satisfied for

$$\begin{aligned} \alpha = \min \left\{ \frac{2}{5} (3 \tau -1), 3(1-2 \tau ) \cdot \left( \frac{q}{5} \right) ^{1/3} \right\} . \end{aligned}$$

Therefore, the best choice of $\tau$ is as follows:

4.7 $$\begin{aligned} \tau _{\#} = \frac{1}{2} - \frac{1}{6 (1+5\kappa )}, \qquad \kappa = \left( \frac{q}{5} \right) ^{1/3}. \end{aligned}$$

In this case, $\alpha _{\#} = \frac{2}{5} (3 \tau _{\#} -1) = \frac{\kappa }{1 + 5 \kappa }$. Thus, we have proved the following theorem.

### Theorem 5

Let the sequence of points $\{ x_k \}_{k \ge 0}$ be generated by the method (4.4) with parameter $\tau = \tau _{\#}$. Then, for all $k \ge 0$, we have

$$\begin{aligned} F(x_k) - F^*\le (1 - \alpha _{\#})^k(F(x_0) - F^*). \end{aligned}$$

In order to generate an $\varepsilon$-solution of problem (2.1) in function value, method (4.4) needs at most

4.8 $$\begin{aligned} \left( 5 + \left( \frac{5}{q}\right) ^{1/3} \right) \ln \frac{F(x_0) - F^*}{\varepsilon } \end{aligned}$$

iterations. Note that for $q = 1$, we have $\alpha _{\#} = \frac{1}{5 + 5^{1/3}} > 0.149$, which is quite close to $\alpha _*$. Thus, the general rule (4.7) is not much worse than the optimal specific rule (3.8).

For practical applications, it is reasonable to use the Euclidean norm $\Vert \cdot \Vert$. In this case, the complexity of one iteration of method (4.4) is the same as that of the usual Cubic Newton Method [22] (see discussion in Example 1).

## Quartic Regularity for Convex Functions

Let us check to what extent the results of the previous sections can be propagated onto the class of general convex functions. Consider the convex unconstrained minimization problem

5.1 $$\begin{aligned} F^*= \min _{x \in \textrm{dom}\,\psi } \left[ F(x) = f(x) + \psi (x)\right] , \end{aligned}$$

where $\psi (\cdot )$ is a simple closed convex function, and the smooth part $f(\cdot )$ of the objective satisfies an additional assumption of *quartic regularity*.

### Definition 1

Function $f(\cdot )$ with convex and open domain $\textrm{dom}\,f \subseteq \mathbb {E}$ is called *quartic regular* (Q-regular) on convex set *S*, if it is four times continuously differentiable on $\textrm{dom}\,f$, and there exist two parameters $L \ge \mu \ge 0$ such that

5.2 $$\begin{aligned} D^4f(x)[h]^4\ge &   \mu \Vert h \Vert ^4, \quad \forall h \in \mathbb {E},~ x \in S, \end{aligned}$$

5.3 $$\begin{aligned} D^4f(x)[h]^4\le &   L \Vert h \Vert ^4, \quad \forall h \in \mathbb {E},~x \in S_{1/2}, \end{aligned}$$

where $$S_{1/2} = \{ z = x - \frac{1}{2} (y-x): \; x, y \in S \} \subseteq \textrm{dom}\,f$$.

This definition can be applied to general norms.^2^

Note that inequality (5.2) alone does not imply convexity of $f(\cdot )$. Thus, for this section, our assumption is as follows.

### Assumption 1

Smooth part of the objective function in problem (5.1) is quartic regular on the set $S \supseteq \textrm{dom}\,\psi$.

Let us mention some trivial properties of Q-regular functions.

- If functions $f_i(\cdot )$ are Q-regular on sets $S_i$ with parameters $(\mu _i,L_i)$, $i=1,2$, then their weighted sum $f(x) = \alpha f_1(x) + \beta f_2(x)$, $\alpha , \beta \ge 0$, is Q-regular on $S_1 \bigcap S_2$ with parameters
  $$ (\alpha \mu _1 + \beta \mu _2, \alpha L_1 + \beta L_2). $$
- If function $\phi (\cdot )$ is Q-regular on $S_1$ with parameters $(\mu ,L)$ and $A: \mathbb {E} \rightarrow \mathbb {E}_1$ is a non-degenerate linear operator, then function $f(x) = \phi (Ax)$ is Q-regular on $S = \{x: \; A x \in S_1 \}$ with parameters $(\mu \sigma ^4_{\min }(A), L\sigma ^4_{\max }(A))$.
- In particular, if function $f(\cdot )$ is Q-regular on *S* with parameters $(\mu ,L)$, then function $\varphi _{\tau }(x) = f(\tau x)$, with scaling factor $\tau \ne 0$, is Q-regular on $\tau ^{-1}S$ with parameters $(\mu \tau ^4, L \tau ^4)$.
- Function $d(x) = \frac{1}{24} \Vert x\Vert ^4$, defined by a Euclidean norm, is Q-regular on $\mathbb {E}$ with parameters $\mu = L = 1$.

The abilities of optimization methods in solving the problem (5.1) are supported by the following inequalities.

### Theorem 6

Let function $f(\cdot )$ be Q-regular on *S* with parameters $\mu$ and *L*. Then for all $x, y \in S$ and $\gamma \in \left[ \frac{1}{3}, \frac{1}{2} \right]$, we have

5.4 $$\begin{aligned} f(y)\ge &   f(x) + \langle \nabla f(x), y - x \rangle + \frac{3 \gamma - 1}{6 \gamma } \langle \nabla ^2 f(x)(y-x), y - x \rangle \nonumber \\  &   + \frac{\mu }{24} \Vert y - x\Vert ^4 \left[ (1-2 \gamma ) - \frac{16}{125} \left( \frac{L}{\mu }-1\right) \left( \frac{3 \gamma - 1}{\gamma } \right) ^3 \right] . \end{aligned}$$

Moreover, for all $\gamma > 0$, we have

5.5 $$\begin{aligned} f(y) \le f(x) + \langle \nabla f(x), y - x \rangle + \frac{3 \gamma + 1}{6 \gamma } \langle \nabla ^2 f(x)(y-x), y - x \rangle + \frac{2 \gamma + 1}{24} L \Vert y - x\Vert ^4. \end{aligned}$$

### Proof

By the Taylor formula, we have

$$\begin{aligned} \varDelta (x,y)  &   \overset{\text {def}}{=} f(y) - f(x) - \langle \nabla f(x), y - x \rangle - \frac{1}{2} \langle \nabla ^2 f(x)(y-x), y - x \rangle \\  &   = \frac{1}{6} D^3f(x)[y-x]^3 + \frac{1}{3!} \int _0^1 (1-\tau )^3 D^4f(x+\tau (y-x)) [y-x]^4 d \tau . \end{aligned}$$

Since $f(\cdot )$ is convex,

$$\begin{aligned} 0 \preceq \nabla ^2 f(x + \gamma (y-x))= &   \nabla ^2 f(x) + \gamma D^3f(x)(y - x) \\  &   + \int _0^\gamma (\gamma - \tau ) D^4 f(x+\tau (y-x))[y-x]^2 d \tau . \end{aligned}$$

Hence,

5.6 $$\begin{aligned} \varDelta (x,y)\ge &   - \frac{1}{6 \gamma } \langle \nabla ^2 f(x)(y-x), y - x \rangle \nonumber \\  &   + \int _0^{\gamma } \left[ \frac{(1 - \tau )^3}{3!} - \frac{1}{6}\left( 1 - \frac{\tau }{\gamma } \right) \right] D^4 f(x+\tau (y-x))[y-x]^4 d \tau \\  &   + \frac{1}{3!} \int _{\gamma }^1 (1-\tau )^3 D^4f(x+\tau (y-x)) [y-x]^4 d \tau .\nonumber \end{aligned}$$

Denote $\theta (\tau ) = (1-\tau )^3 + \frac{\tau }{\gamma } - 1 = \tau \left( \frac{1}{\gamma } - 3 \right) + 3 \tau ^2 - \tau ^3$. This cubic polynomial has three real roots, one at $\tau = 0$ and two others at the points

$$ \tau _{0,1}(\gamma ) = \frac{3}{2} \pm \sqrt{\frac{1}{\gamma }- \frac{3}{4}}. $$

Note that by conditions of the theorem, we have

$$ 0 \le \tau _0(\gamma ) \le \gamma . $$

Therefore, at the interval $\tau \in [0,1]$, we have the following relations:

$$ \theta (\tau ) \le 0, \quad \tau \in [0,\tau _{0}(\gamma )], \quad \theta (\tau ) \ge 0, \quad \tau _0(\tau ) \le \tau \le 1 \quad (\le \tau _1(\gamma )). $$

Denoting the first integral in (5.6) by $I_1$, the second one by $I_2$, and $r = \Vert y - x\Vert$, we have

$$\begin{aligned} 6 I_1= &   \int _0^{\tau _0(\gamma )} \theta (\tau ) D^4 f(x+\tau (y-x))[y-x]^4 d \tau \\    &   + \int _{\tau _0(\gamma )}^{\gamma } \theta (\tau ) D^4 f(x+\tau (y-x))[y-x]^4 d \tau \\\overset{(5.3)}{\ge } &   L r^4 \int _0^{\tau _0(\gamma )} \theta (\tau ) d \tau + \mu r^4 \int _{\tau _0(\gamma )}^{\gamma } \theta (\tau ) d \tau \\= &   (L-\mu ) r^4 \int _0^{\tau _0(\gamma )} \theta (\tau ) d \tau + \mu r^4 \int _{0}^{\gamma } \theta (\tau ) d \tau . \end{aligned}$$

On the other hand, $6 I_2 \overset{(5.2)}{\ge } \mu r^4 \int _{\gamma }^1 (1-\tau )^3 d \tau$. Note that

$$ \int _{0}^{\gamma } \theta (\tau ) d \tau + \int _{\gamma }^1 (1-\tau )^3 d \tau = \int _{0}^1 (1-\tau )^3 d \tau + \int _0^{\gamma } \left( \frac{\tau }{\gamma } - 1\right) d \tau = \frac{1 - 2 \gamma }{4} $$

and we conclude that

$$ 6(I_1+I_2) \ge (L-\mu ) r^4 \int _0^{\tau _0(\gamma )} \theta (\tau ) d \tau + \mu r^4 \frac{1 - 2 \gamma }{4}. $$

It remains to estimate the integral in the latter inequality from below.

Denoting for short $\tau _0 = \tau _0(\gamma )$, we have

$$\begin{aligned} I_0  &   \overset{\text {def}}{=} \int _0^{\tau _0} \theta (\tau ) d \tau = \int _0^{\tau _0} \left[ (1-\tau )^3 + \frac{\tau }{\gamma } - 1 \right] d \tau \\  &   = - \frac{1}{4}(1-\tau )^4 + \frac{\tau ^2}{2 \gamma } - \tau \Big |_0^{\tau _0} = - \frac{1}{4}(1-\tau _0)^4 + \frac{\tau _0^2}{2 \gamma } - \tau _0 + \frac{1}{4}. \end{aligned}$$

Since $\theta (\tau _0) = 0$, we conclude that

$$ I_0 = -\frac{1}{4}(1-\tau _0)\left( 1 - \frac{\tau _0}{\gamma } \right) + \frac{\tau _0^2}{2 \gamma } - \tau _0 + \frac{1}{4} = - \frac{3 \gamma - 1}{4 \gamma } \tau _0 + \frac{\tau _0^2}{4 \gamma }. $$

Denote $\xi = \frac{3 \gamma - 1}{\gamma }$ and $\lambda = \frac{4}{9} \xi$. Then $0 \le \xi \le 1$, $0 \le \lambda \le \frac{4}{9}$, and

$$ \tau _0 = \frac{3}{2} - \sqrt{\frac{1}{\gamma } - \frac{3}{4}} = \frac{3}{2} - \sqrt{\frac{9}{4} - \xi } = \frac{3}{2} \left( 1 - \sqrt{1 - \lambda }\right) \le \frac{3}{2} \cdot \frac{3}{5} \lambda = \frac{2}{5} \xi . $$

Note that in our new notation we have

5.7 $$\begin{aligned} 3 \tau _0 - \tau _0^2 = 3 - \frac{1}{\gamma } = \xi . \end{aligned}$$

Therefore,

$$\begin{aligned} 4 I_0= &   - \xi \tau _0 + \frac{1}{\gamma } \tau _0^2 \overset{(5.7)}{=} -(3 \tau _0 - \tau _0^2)\tau _0 + \frac{1}{\gamma } \tau _0^2 = \tau _0^3 - \xi \tau _0^2\\\overset{(5.7)}{=} &   \tau _0^3 - (3 \tau _0 - \tau _0^2) \tau _0^2 = - 2 \tau _0^3 + \tau _0^4 \ge - 2 \tau _0^3 \ge -\frac{16}{125} \xi ^3. \end{aligned}$$

Thus, we get the following lower bound:

$$\begin{aligned} f(y) - f(x) - \langle \nabla f(x), y - x \rangle\ge &   \frac{\xi }{6} \langle \nabla ^2 f(x)(y-x), y - x \rangle \\  &   + \frac{\mu r^4}{6} \left[ \frac{1 - 2 \gamma }{4} - \frac{4}{125} \left( \frac{L}{\mu }-1\right) \xi ^3 \right] . \end{aligned}$$

In order to prove the upper bound, note that

$$\begin{aligned} 0 \preceq \nabla ^2 f(x - \gamma (y-x))= &   \nabla ^2 f(x) - \gamma D^3f(x)(y - x) \\  &   + \int _0^\gamma (\gamma - \tau ) D^4 f(x-\tau (y-x))[y-x]^2 d \tau . \end{aligned}$$

Hence,

$$\begin{aligned} \varDelta (x,y)\le &   \frac{1}{6 \gamma } \langle \nabla ^2 f(x)(y-x), y - x \rangle \\  &   + \frac{1}{6 \gamma } \int _0^{\gamma }(\gamma - \tau ) D^4 f(x-\tau (y-x))[y-x]^4 d \tau \\  &   + \frac{1}{3!} \int _{0}^1 (1-\tau )^3 D^4f(x+\tau (y-x)) [y-x]^4 d \tau \\\overset{(5.3)}{\le } &   \frac{1}{6 \gamma } \langle \nabla ^2 f(x)(y-x), y - x \rangle + \frac{L}{6} \Vert y - x \Vert ^4 \left( \frac{\gamma }{2} + \frac{1}{4} \right) . \end{aligned}$$

$$\square $$

Now we can estimate performance of method (4.4) as applied to problem (5.1). Define

$$\begin{aligned} \hat{\xi }_{x,\gamma }(y)&\overset{\text {def}}{=}&f(x) + \langle f(x), y - x \rangle + \frac{1 + 3\gamma }{6 \gamma } \langle \nabla ^2 f(x) (y-x), y - x \rangle \\  &   + \frac{1+2\gamma }{24} L \Vert y - x \Vert ^4 \overset{(5.5)}{\ge } f(y), \quad x, y \in S, \end{aligned}$$

where $f(\cdot )$ is a Q-regular function. Consider the following optimization method.

5.8 $$\begin{aligned} \begin{array}{c} {\textbf {Quartic Regularization of Newton Method}}\\ {\textbf {for Q-Regular Functions}}\\ \\ \\ \begin{array}{l} {{\textbf {Initialization.}}\,\, \textrm{Choose}\, x_0 \in \textrm{dom}\,\psi \, \textrm{and}\, \textrm{parameter}\, \gamma > 0.}\\ \\ {{ k {\textbf {th iteration}}\, (k \ge 0).}\,\, \textrm{Iterate}\, x_{k+1} = \arg \min \limits _{y \in \textrm{dom}\,\psi } \left[ \hat{\xi }_{x_k, \gamma }(y) + \psi (y)\right] .}\\ \\ \end{array} \end{array} \end{aligned}$$

### Theorem 7

Let function $f(\cdot )$ be Q-regular on the sets $S \supseteq \textrm{dom}\,\psi$ with parameters $0 < \mu \le L$. And let the parameter $\gamma \in \left( \frac{1}{3}, \frac{1}{2} \right)$ of method (5.8) satisfy the following condition:

5.9 $$\begin{aligned} \kappa (\gamma )  &   \overset{\text {def}}{=} (1-2 \gamma ) - \frac{16}{125} \left( \frac{L}{\mu }-1\right) \left( \frac{3 \gamma - 1}{\gamma } \right) ^3 \ge 0,\\ \alpha  &   \le \min \left\{ \frac{3 \gamma - 1}{3 \gamma + 1}, \left[ \frac{\mu }{L(1+2\gamma )} \kappa (\gamma ) \right] ^{1/3} \right\} \nonumber \end{aligned}$$

for some $\alpha > 0$. Then, for all $k \ge 0$, we have

5.10 $$\begin{aligned} F(x_k) - F^*\le (1 - \alpha )^k(F(x_0) - F^*). \end{aligned}$$

### Proof

As in the proof of Theorem 4, define the following parametric functions:

$$\begin{aligned} \hat{\xi }_{x,\gamma }(\alpha ,y)= &   f(x) + \langle \nabla f(x), y - x \rangle + \frac{3 \gamma - 1}{6 \alpha \gamma } \langle \nabla ^2 f(x)(y-x), y - x \rangle \\  &   +\frac{\mu }{24 \alpha ^3} \Vert y - x\Vert ^4 \kappa (\gamma ),\\ \Xi ^*_{x,\gamma }(\alpha )= &   \min _{y \in \textrm{dom}\,\psi } \left[ \hat{\xi }_{x,\gamma }(\alpha ,y) + \psi (y)\right] , \quad \alpha > 0. \end{aligned}$$

Note that $\Xi ^*_{x,\gamma }(0) = F(x)$ and $\hat{\xi }_{x,\gamma }(1,y) \overset{(5.4)}{\le } f(y)$. Thus, $\Xi ^*_{x,\gamma }(1) \le F^*$. On the other hand, in view of conditions (5.9), we have

$$ \frac{3 \gamma - 1}{6 \alpha \gamma } \ge \frac{3 \gamma +1}{6 \gamma }, \quad \frac{\mu }{24 \alpha ^3} \kappa (\gamma ) \ge \frac{1 + 2 \gamma }{24}L. $$

Therefore, as in the proof of Theorem 4, we conclude that

$$ F(x_{k+1}) \le \Xi ^*_{x,\gamma }(\alpha ) \le \alpha F^*+ (1-\alpha ) F(x_k). $$

$$\square $$

Note that method (5.8) can be endowed with a termination criterion, which supports its rate of convergence (5.10). Indeed, in this method, in parallel with the minimizing sequence $\{x_k\}_{k \ge 0}$, we can update the following lower bounds for the optimal value of problem (5.1):

5.11 $$\begin{aligned} \xi ^*_0 = \Xi ^*_{x_0,\gamma }(1), \quad \xi ^*_{k+1} = \max \left\{ \xi ^*_k, \Xi ^*_{x_k,\gamma }(1) \right\} , \quad k \ge 0. \end{aligned}$$

Clearly, under conditions of Theorem 7, for all $k \ge 0$, we have

5.12 $$\begin{aligned} \text {(a):} ~\xi ^*_k \le \xi ^*_{k+1} \le F^*, \qquad \text {(b):} ~ \xi _{k+1}^*\ge \Xi ^*_{x_k,\gamma }(1). \end{aligned}$$

### Theorem 8

Let the conditions of Theorem 7 be satisfied. Then, for all $k \ge 0$, we have

5.13 $$\begin{aligned} F(x_k) - F^*\le F(x_k) - \xi _k^*\le (1-\alpha )^k (F(x_0) - \xi ^*_0). \end{aligned}$$

### Proof

Indeed, by the same reasons as in the proof of Theorem 4, we can see that

$$\begin{aligned} F(x_{k+1}) - \xi ^*_{k+1}\le &   \alpha \Xi ^*_{x_k,\gamma }(1) + (1-\alpha )F(x_k) - \xi ^*_{k+1} \\\overset{(5.12\mathrm b)}{\le } &   (1-\alpha )\left( F(x_k) - \xi ^*_{k+1}\right) \overset{(5.12\mathrm a)}{\le } (1-\alpha )(F(x_k) - \xi ^*_{k}). \end{aligned}$$

$$\square $$

Thus, if the inequality

$$\begin{aligned} F(x_k) - \xi _k^*\le \varepsilon \end{aligned}$$

is satisfied, then the point $x_k$ is an $\varepsilon$-solution of problem (5.1).

Finally, let us provide method (5.8) with a reasonable value of parameter $\gamma$. In accordance to the relation (5.9), it could be found from the balance equation

$$ \left( \frac{3 \gamma - 1}{3 \gamma + 1} \right) ^3 = \frac{\mu \kappa (\gamma )}{L(1+2\gamma )}. $$

However, it is quite complicated. Moreover, we need to emphasize the asymptotic dependence of the coefficient $\alpha$ of (5.9) in the condition number *q*. Therefore, we are going to use an approximate solution of the above equation.

### Theorem 9

Let $q = \frac{\mu }{L} \le 1$. For coefficient

5.14 $$\begin{aligned} \gamma = \gamma _*\overset{\text {def}}{=} \frac{1}{3\left( 1 - \frac{3}{11}q^{1/3}\right) } \le \frac{11}{24}, \end{aligned}$$

we have

5.15 $$\begin{aligned} \frac{3 \gamma - 1}{3 \gamma + 1} \le \left[ \frac{q\kappa (\gamma )}{1+2\gamma }\right] ^{1/3}. \end{aligned}$$

### Proof

Let $\gamma = \gamma _*\overset{\text {def}}{=} \frac{1}{3(1 - \tau _*q^{1/3})}$ with $\tau _*= \frac{3}{11}$. Denote $\lambda = \frac{3 \gamma - 1}{3 \gamma } = \tau _*q^{1/3}$. We need to prove that

$$\begin{aligned} (1+2\gamma ) \left( \frac{3 \gamma - 1}{3 \gamma + 1}\right) ^3\le &   q \left( (1-2 \gamma ) - \frac{16}{125} \left( q^{-1}-1\right) \left( \frac{3 \gamma - 1}{\gamma } \right) ^3 \right) \\= &   q \left( (1-2 \gamma ) + 16 \left( \frac{3}{5}\right) ^3 \lambda ^3 \right) - 16 \left( \frac{3}{5}\right) ^3 \lambda ^3. \end{aligned}$$

Since $\gamma \le \frac{1}{2}$, we have $\left( \frac{3 \gamma - 1}{3 \gamma + 1}\right) ^3 = \left( \frac{3 \gamma }{3 \gamma + 1}\right) ^3 \lambda ^3 \le \left( \frac{3}{5} \right) ^3 \lambda ^3$. Thus, it is enough to prove that

$$ \left[ (1 + 2 \gamma ) \left( \frac{3}{5} \right) ^3 + 16 \left( \frac{3}{5} \right) ^3 \right] \lambda ^3 \le q (1 - 2 \gamma + 16 \lambda ^3). $$

However, $\lambda ^3 = O(q)$. Hence, we can relax our goal up to the following target inequality:

5.16 $$\begin{aligned} 18 \left( \frac{3}{5} \right) ^3\lambda ^3 \le \frac{q}{12} \quad \left( \overset{(5.14)}{\le } q (1 - 2 \gamma ) \right) . \end{aligned}$$

By the definition of $\lambda$, this is the inequality $18 \left( \frac{9}{55} \right) ^3 \le \frac{1}{12}$, which is valid since $18 \cdot 12 = 6^3$.

Thus, inequality (5.16) is valid and we prove the relation (5.15). $\square$

Now, using $\gamma = \gamma _*$ in inequality (5.4), and denoting $\tau _*= \frac{3}{11}$, we get the following lower bound:

$$\begin{aligned}  &   f(y) - f(x) - \langle \nabla f(x), y - x \rangle \\  &   \qquad \ge \frac{3 q^{1/3}}{22} \langle \nabla ^2 f(x)(y-x), y - x \rangle + \frac{L (1+2 \gamma _*)}{24} \Vert y - x\Vert ^4 \left( \frac{3 \gamma _*- 1}{3 \gamma _*+ 1} \right) ^3\\  &   \qquad \ge \frac{3 q^{1/3}}{22} \langle \nabla ^2 f(x)(y-x), y - x \rangle + \frac{5 \mu }{3 \cdot 24} \Vert y - x \Vert ^4 \left( \frac{\tau _*}{2 - \tau _*q^{1/3}} \right) ^3\\  &   \qquad \ge \frac{3 q^{1/3}}{22} \langle \nabla ^2 f(x)(y-x), y - x \rangle + \frac{5 \mu }{3 \cdot 24} \left( \frac{3}{22} \right) ^3 \Vert y - x \Vert ^4. \end{aligned}$$

Relation (5.15) can be also used for justifying the rate of convergence of method (5.8). Indeed, if we choose in (5.8) $\gamma = \gamma _*$, then in accordance to Theorems 7 and 9, the rate of convergence (5.10) is described by

$$ \alpha = \alpha _*\overset{\text {def}}{=} \frac{3 \gamma _*- 1}{3 \gamma _*+ 1}. $$

Note that

$$ \alpha _*\overset{(5.14)}{=} \frac{3q^{1/3}}{22 - 3 q^{1/3}}. $$

Thus, in order to get $\varepsilon$-solution of problem (2.1), the pure second-order method (5.8) with $\gamma = \gamma _*$ needs

5.17 $$\begin{aligned} \frac{22}{3 q^{1/3}} \ln \frac{F(x_0) - F^*}{\varepsilon } \end{aligned}$$

iterations at most. Note that this bound is approximately in four times worse than the bound (4.8) for efficiency of a specialized method (4.4) as applied to the quartic polynomials.

## Applications in Convex Optimization

Let us show now that the notion of Q-regularity can be used for constructing new and efficient optimization schemes. In this section, we are working with Euclidean norm $\Vert x\Vert = \langle B x, x \rangle ^{1/2}$ defined by a self-adjoint linear operator $B \succ 0$.

Consider first the problem (5.1) with bounded fourth derivative of function $f(\cdot )$:

$$\begin{aligned} 0 \le D^4f(x)[h]^4 \le L \Vert h\Vert ^4, \quad \forall x \in S,~h \in \mathbb {E}. \end{aligned}$$

Since $\mu = 0$, such a problem cannot be solved directly by method (5.8). However, we can treat it by an appropriate regularization technique.

Indeed, let $\varepsilon >0$ be our target accuracy in function value for an approximate solution of problem (5.1). Suppose that we know an upper bound for the distance to the optimal solution:

6.1 $$\begin{aligned} \Vert x_0 - x^*\Vert \le R. \end{aligned}$$

Then we can define the following regularized version of the objective function:

$$\begin{aligned} F_{x_0,H}(x)  &   \overset{\text {def}}{=} F(x) + \frac{H}{24} \Vert x - x_0\Vert ^4, \quad x \in \textrm{dom}\,\psi ,\\ H  &   = \frac{12\varepsilon }{R^4}. \end{aligned}$$

Note that this function is Q-regular with the following parameters:

$$\begin{aligned} \mu _{\varepsilon } = H, \quad L_{\varepsilon } = L + H, \quad q_{\varepsilon }^{-1} = 1 + \frac{LR^4}{12 \varepsilon }. \end{aligned}$$

Hence, since $F_{x_0,H}(x_0) = F(x_0)$ and $F^*_{x_0,H} \ge F^*$, inequality (5.17) provides us with the following bound on the number of iterations of method (5.8) needed for finding an $(\varepsilon /2)$-solution of the regularized problem:

$$\begin{aligned} \frac{22}{3} \left[ 1 + \frac{LR^4}{12 \varepsilon } \right] ^{1/3} \ln \frac{2 (F(x_0) - F^*)}{\varepsilon }. \end{aligned}$$

Let $\bar{x}$ be such a point, and $x^*$ be an optimal solution of problem (5.1). Then

$$\begin{aligned} F(\bar{x})\le &   F_{x_0,H}(\bar{x}) \le F^*_{x_0,H} + \frac{\varepsilon }{2} \le F_{x_0,H}(x^*) + \frac{\varepsilon }{2} \\= &   F(x^*) + \frac{\varepsilon }{2 R^4} \Vert x^*- x_0 \Vert ^4 + \frac{\varepsilon }{2} \overset{(6.1)}{\le } F^*+ \varepsilon . \end{aligned}$$

Thus, the point $\bar{x}$ is an $\varepsilon$-solution of our initial problem (5.1).

However, the notion of Q-regularity finds much more important applications in the framework of high-order proximal-point methods [2, 21]. In this approach, we solve the general problems of composite minimization in the form

6.2 $$\begin{aligned} F^*= \min _{x \in \textrm{dom}\,\psi } \left[ F(x) = f(x) + \psi (x)\right] , \end{aligned}$$

where $\psi (\cdot )$ is a simple closed convex function, and the smooth part $f(\cdot )$ of the objective function is convex and has bounded fourth derivative:

6.3 $$\begin{aligned} |D^4f(x)[h]^4|\le M_4 \Vert h \Vert ^4, \quad \forall x \in (\textrm{dom}\,\psi )_{1/2},~ h \in \mathbb {E}. \end{aligned}$$

The problem (6.2) can be solved by different third-order proximal-point methods [20, 21], provided that we are able to compute in a reasonable time an approximate solution to the following auxiliary optimization problems:

6.4 $$\begin{aligned} \min _{x \in \textrm{dom}\, \psi } \left[ F_{\bar{x}, H}(x) = F(x) + \frac{H}{24} \Vert x - \bar{x} \Vert ^4\right] \end{aligned}$$

with different prox-centers $\bar{x} \in \textrm{dom}\,\psi$. Note that the objective function of this problem can be represented as follows:

$$\begin{aligned} F_{\bar{x}, H}(x)= &   f_{\bar{x}, H}(x) + \psi (x),\\ f_{\bar{x}, H}(x)= &   f(x) + \frac{H}{24} \Vert x - \bar{x} \Vert ^4. \end{aligned}$$

Hence, for *H* big enough, the smooth part of the objective function in (6.4) is *Q*-regular.

### Lemma 5

Let convex function $f(\cdot )$ satisfy condition (6.3). Then for $H \ge M_4$, function $f_{\bar{x},H}(\cdot )$ is Q-regular with parameters $\mu = H-M_4$ and $L = H+M_4$.

### Proof

Indeed, since the norm $\Vert \cdot \Vert$ is Euclidean, for function $d(x) = \frac{1}{24} \Vert x \Vert ^4$ we have

$$ D^4d(x)[h]^4 = \Vert h \Vert ^4, \quad \forall x, h \in \mathbb {E}. $$

It remains to use inequality (6.3). $\square$

Thus, for *H* big enough, problem (6.4) can be efficiently solved by the Quartic Newton Method (5.8). This method has linear rate of convergence in function value. Let us find an upper bound for the number of iterations, which is sufficient for finding an appropriate approximate solution of problem (6.4).

We need two auxiliary results. Let $\varPhi (x) = \varphi (x) + \psi (x)$, where $\psi (\cdot )$ is a simple closed convex function, and function $\varphi (\cdot )$ is twice continuously differentiable on $\textrm{dom}\,\psi$. Consider the following problem of composite minimization:

$$ \varPhi _*= \min _{x \in \textrm{dom}\,\psi } \varPhi (x). $$

### Lemma 6

Let us fix a point $\bar{x} \in \textrm{dom}\,\psi$ with $\varPhi (\bar{x}) > \varPhi _*$. Define the point

6.5 $$\begin{aligned} T = T(\bar{x}) \overset{\text {def}}{=} \arg \min _{x \in \textrm{dom}\, \psi } \left[ \langle \nabla \varphi (\bar{x}), x \rangle + \frac{M}{2} \Vert x - \bar{x} \Vert ^2 + \psi (x)\right] , \end{aligned}$$

where $M \ge \hat{M} = \sup _{x \in \mathcal {F}} \Vert \nabla ^2 \varphi (x) \Vert$ and $\mathcal {F} = \{x \in \textrm{dom}\,\psi : \varPhi (x) \le \varPhi (\bar{x}) \}$. Then

6.6 $$\begin{aligned} \varPhi '(T)  &   \overset{\text {def}}{=} \nabla \varphi (T) - \nabla \varphi (\bar{x}) - M B(T-\bar{x}) \in \partial \varPhi (T),\\ (\Vert \varPhi '(T) \Vert ^*)^2  &   \le 2M [\varPhi (\bar{x}) - \varPhi (T)].\nonumber \end{aligned}$$

### Proof

Consider the point $\hat{T} = \arg \min _{x \in \mathcal {F}} \left[ \langle \nabla \varphi (\bar{x}), x \rangle + \frac{M}{2} \Vert x - \bar{x} \Vert ^2 + \psi (x)\right]$. The first-order optimality condition for this problem is as follows:

6.7 $$\begin{aligned} \langle \nabla \varphi (\bar{x}) + MB(\hat{T} - \bar{x}), x - \hat{T} \rangle + \psi (x) \ge \psi (\hat{T}), \quad \forall x \in \mathcal {F}. \end{aligned}$$

If $\hat{T} = \bar{x}$, then for $x_*\in \text {Arg}\min _{\textrm{dom}\,\psi } \varPhi (x)$, we have

$$ \varPhi (x_*) = \varphi (x_*) + \psi (x_*) \ge \varphi (\bar{x}) + \langle \nabla \varphi (\bar{x}), x_*- \bar{x} \rangle + \psi (x_*) \overset{(6.7)}{\ge } \varPhi (\bar{x}), $$

and this contradicts to the assumptions of the theorem.

Thus, $\hat{T} \ne \bar{x}$. Applying (6.7) to $x = \bar{x}$ and since both $\bar{x}$ and $\hat{T}$ belong to $\mathcal {F}$, we have

$$\begin{aligned} \varPhi (\hat{T})= &   \varphi (\hat{T}) + \psi (\hat{T})\\\le &   \varphi (\bar{x}) + \langle \nabla \varphi (\bar{x}), \hat{T} - \bar{x} \rangle + \frac{M}{2} \Vert \hat{T} - \bar{x}\Vert ^2 + \psi (\hat{T})\\\overset{(6.7)}{\le } &   \varPhi (\bar{x}) - \frac{M}{2} \Vert \hat{T} - \bar{x} \Vert ^2 < \varPhi (\bar{x}). \end{aligned}$$

Hence, the functional boundary of the level set $\mathcal {F}$ is not active at $\hat{T}$ and therefore $\hat{T} = T$ as defined by (6.5).

Note that the first-order optimality condition for problem (6.5) is as follows:

$$\begin{aligned} \langle \nabla \varphi (\bar{x}) + MB(T - \bar{x}), x - T \rangle + \psi (x) \ge \psi (T), \quad \forall x \in \textrm{dom}\,\psi . \end{aligned}$$

This means that $\psi '(T) \overset{\text {def}}{=} - \nabla \varphi (\bar{x}) - MB(T - \bar{x}) \in \partial \psi (T)$ and the inclusion

$$ \varPhi '(T) = \nabla \varphi (T) + \psi '(T) \in \partial \varphi (T) $$

is valid. On the other hand, since both $\bar{x}$ and *T* belong to $\mathcal {F}$, we conclude that

$$\begin{aligned} (\Vert \varPhi '(T)\Vert ^*)^2= &   (\Vert \varphi (T) - \nabla \varphi (\bar{x}) - MB(T - \bar{x}) \Vert ^*)^2\\= &   (\Vert \varphi (T) - \nabla \varphi (\bar{x})\Vert ^*)^2 - 2 M \langle \varphi (T) - \nabla \varphi (\bar{x}), T - \bar{x} \rangle + M^2 \Vert T - \bar{x} \Vert ^2\\\overset{(1.2)}{\le } &   M^2 \Vert T - \bar{x} \Vert ^2. \end{aligned}$$

This gives us the bound (6.6). $\square$

We are going to apply Lemma 6 in the situation where the function $\varphi (\cdot )$ is Q-regular. Hence, we need to use an explicit bound for its second derivative.

### Lemma 7

Let function $\varphi (\cdot )$ be Q-regular on $\textrm{dom}\,\psi$ with constants $L \ge \mu > 0$. Then, for any $x \in \mathcal {F} = \{ x \in \textrm{dom}\,\psi : \; \varPhi (x) \le \varPhi (\bar{x}) \}$ we have

$$\begin{aligned} \Vert \nabla ^2 \varphi (x) \Vert \le \hat{M} \overset{\text {def}}{=} 4 \Vert \nabla ^2 \varphi (\bar{x}) \Vert + \frac{2}{3} LD^2, \end{aligned}$$

where $$D \overset{\text {def}}{=} 2 \left[ \frac{72}{\mu } (\varPhi (\bar{x}) - \varPhi _*)\right] ^{1/4}$$.

### Proof

Since $\mu > 0$, function $\varPhi (\cdot )$ is uniformly convex. Let us fix $\gamma = \frac{1}{3}$. By inequality (5.4), we get

$$ \frac{\mu }{72} \Vert \bar{x} - x_*\Vert ^4 \le \varPhi (\bar{x}) - \varPhi (x_*) \overset{\text {def}}{=} \varDelta , $$

where $x_*= \arg \min _{x \in \textrm{dom}\,\psi } \varPhi (x)$. Thus, $\Vert \bar{x} - x_*\Vert \le R \overset{\text {def}}{=} \left[ \frac{72}{\mu } \varDelta \right] ^{1/4}$. Note that the same bound on the distance to the optimum is valid for any point from the level set $\mathcal {F}$. Hence, for any $y \in \mathcal {F}$ we have $\Vert y - \bar{x} \Vert \le D = 2 R$.

In view of (5.3), for any $y \in \textrm{dom}\,\psi$ we have

$$ 0 \preceq \nabla ^2 \varphi (\bar{x} - \gamma (y-\bar{x})) \preceq \nabla ^2 \varphi (\bar{x}) - \gamma D^3\varphi (\bar{x})[y - \bar{x}] + \frac{\gamma ^2}{2} L \Vert y - \bar{x} \Vert ^2 B. $$

Therefore, for any $y \in \mathcal {F}$, we get the following bound:

$$\begin{aligned} \nabla ^2 \varphi (y)\preceq &   \nabla ^2 \varphi (\bar{x}) + D^3 \varphi (\bar{x})[y-\bar{x}] + \frac{1}{2} L \Vert y - \bar{x} \Vert ^2 B\\\preceq &   \frac{1 + \gamma }{\gamma } \nabla ^2 \varphi (\bar{x}) + \frac{1 + \gamma }{2} L \Vert y - \bar{x} \Vert ^2 B. \end{aligned}$$

Thus, $\hat{M} = \frac{1 + \gamma }{\gamma } \Vert \nabla ^2 \varphi (\bar{x}) \Vert + \frac{1 + \gamma }{2} LD^2$ is an upper bound for the norms of Hessians of function $\varphi (\cdot )$ on the set $\mathcal {F}$. $\square$

Let us show now how we can use all this machinery in the framework of high-order proximal-point methods of degree three. Recall that an approximation $\hat{x} \in \textrm{dom}\,\psi$ of the exact minimum of problem (6.4) is acceptable if it satisfies the following condition:

6.8 $$\begin{aligned} \exists \hat{g} \in \partial \psi (\hat{x}): \quad \Vert \nabla F_{\bar{x}, H}(\hat{x}) + \hat{g} \Vert ^*\le \beta \Vert \nabla f(\hat{x}) + \hat{g} \Vert ^*, \end{aligned}$$

where $\beta \in (0,1)$ is the tolerance parameter (see [2]; compare with [21]). Note that at the exact solution of problem (6.4) we can enforce the left-hand side of this condition to be zero.

Let us estimate complexity of finding such a point by QRNM (5.8). The whole process consists of two stages, dependent on positive parameteres $\delta$ and *M*.

- **Stage 1.** Suppose that we know the constant $M_4$ in (6.3). Then, taking $H = 2M_4$, we can ensure Q-regularity of function $f_{\bar{x},h}(\cdot )$ with parameters
  $$ \mu = M_4, \quad L = 3 M_4, \quad q = \frac{1}{3} $$
  (see Lemma 5). Hence, choosing in (5.8) $\gamma \overset{(5.14)}{=} \gamma _*$, we get the minimizing sequence convergent with the following rate:
  6.9 $$\begin{aligned} F_{\bar{x}, H}(x_k) - \xi _k^*\overset{(5.13)}{\le } (1 - \alpha _*)^k (F(\bar{x}) - F^*), \end{aligned}$$
  where $\xi _k^*$ are computable lower bounds for the optimal value of (6.4) updated in accordance to (5.11). This stage is terminated when $F_{\bar{x}, H}(x_k) - \xi _k^*\le \delta$.
- **Stage 2.** Compute point $\hat{x} = T(x_k)$ using the iteration (6.5) with $\varphi (\cdot ) = f_{\bar{x}, H}(\cdot )$ and an appropriate constant *M*.

Let us point out the values of parameters $\delta$ and *M* ensuring validity of the condition (6.8). In view of Lemma 7, we can take

$$ M = 4 \Vert \nabla ^2 f(\bar{x}) \Vert + 2 D^2, \quad D= 2 \left[ \frac{72}{M_4} (F(\bar{x}) - F^*)\right] ^{1/4}. $$

Thus, we come to the following conclusion.

### Lemma 8

Let problem (6.2) satisfy the following assumptions:

- $$\Vert \nabla ^2 f(x)\Vert \le M_2$$, $$x \in \textrm{dom}\,\psi $$,
- $$F(x) - F(y) \le \varDelta _F$$, $$x, y \in \textrm{dom}\,\psi $$.

Then we can take

$$\begin{aligned} M = 4 M_2 + 48 \left[ \frac{2 \varDelta _F}{M_4}\right] ^{1/2}. \end{aligned}$$

Suppose now that $F(\hat{x}) - F^*\ge \varepsilon$. Then in the condition (6.8) we have

$$ \Vert \nabla f(\hat{x}) + \hat{g}\Vert ^*\ge (F(\hat{x}) - F^*)/ D_{\psi } \ge \varepsilon / D_{\psi }, $$

where $D_{\psi } < + \infty$ is the diameter of $\textrm{dom}\,\psi$. Hence, in view of inequality (6.6), in order to satisfy condition (6.8), it is sufficient to ensure

$$ 2 M (F_{\bar{x}, H}(x_k) - \xi _k^*) \le \left( \frac{\beta \varepsilon }{D_{\psi }} \right) ^2. $$

Taking into account the rate of convergence (6.9), we get the following upper bound for the number of iterations of method (5.8) for solving the auxiliary problem (6.4):

$$\begin{aligned} \frac{22}{3^{2/3}} \ln \frac{2 M \varDelta _F D_{\psi }^2}{\beta ^2 \varepsilon ^2}. \end{aligned}$$

Having this machinery at hand, we can design different second-order methods for solving the problem (6.2) with the rate of convergence $O(k^{-p})$ with $p = 3, 4, 5$ (see [2, 3]).

## Footnotes

1 It is traditionally defined by the uniform lower and upper bounds for the eigenvalues of the Hessian of the objective function. These constants control the growth of the objective with respect to its linear approximation at the current test point.

2 As compared to (4.1), it is more convenient now to work directly with the fourth derivative.
